# Comparison of *KRAS *and *EGFR *gene status between primary non-small cell lung cancer and local lymph node metastases: implications for clinical practice

**DOI:** 10.1186/1756-9966-30-30

**Published:** 2011-03-17

**Authors:** Leina Sun, Qiang Zhang, Huanling Luan, Zhongli Zhan, Changli Wang, Baocun Sun

**Affiliations:** 1 Department of Pathology, Tianjin Medical University Cancer Institute and Hospital; Tianjin 300060, China; 2 Department of Thoracic Surgery, Tianjin Medical University Cancer Institute and Hospital; Tianjin 300060, China; 3 Key Laboratory of Cancer Prevention and Therapy, Tianjin 300060, China; 4 Tianjin Diagnosis and Therapy Center of Lung Cancer, Tianjin 300060, China

## Abstract

**Background:**

Epidermal growth factor receptor tyrosine kinase inhibitors (EGFR-TKI) have been widely used for the treatment of non-small cell lung cancer (NSCLC). *KRAS *and *EGFR *somatic mutations in NSCLC may predict resistance and responsiveness to TKI, respectively. Nevertheless, most research to date has been conducted on samples from primary tumors. For many patients with advanced disease, their samples can only be obtained from metastases for test. The molecular characteristics of metastasized tumors may be different from those of primary tumors.

**Materials and methods:**

Mutation status of *KRAS *and *EGFR *between primary tumors and local lymph node metastases of 80 Chinese patients with NSCLC were analyzed by direct sequencing. Five of them were given gefitinib as neoadjunvant treatment after the EGFR-TKI sensitive mutations were detected in their biopsies of mediastinal lymph nodes metastases. McNemar's test was used to compare the *EGFR *and *KRAS *mutation status between primary tumors and corresponding local lymph node metastases. Data evaluation was carried out with SPSS_13.0 statistical software.

**Results:**

Among the 160 samples, one primary tumor and seven metastases were identified with *KRAS *mutations and 21 primary tumors and 26 metastases were found to have *EGFR *mutations. *KRAS *and *EGFR *mutation status was different between primary tumors and corresponding metastases in 6 (7.5%) and 7 (8.75%) patients, respectively. One patient with no TKI sensitive mutations detected in the primary tumor showed disease progression.

**Conclusion:**

Our results suggest that a considerable proportion of NSCLC in Chinese population showed discrepancy in *KRAS *and *EGFR *mutation status between primary tumors and corresponding metastases. This observation may have important implication for the use of targeted TKI therapy in the treatment of NSCLC patients.

## Introduction

Lung cancer is one of the leading causes of cancer-related mortality both in China and throughout the world [1,2]. Non-small cell lung cancer (NSCLC) accounts for75-80% of all lung cancer [3]. Standard therapeutic strategies such as surgery, chemotherapy, or radiotherapy have reached a plateau [1]. Significant advances in the research of the biology and molecular mechanisms of cancer have allowed the development of new molecularly targeted agents for the treatment of NSCLC [4-8]. One such target is the epidermal growth factor receptor (EGFR), a 170-kDa trans-membrane glycoprotein and member of erbB family. Small molecule tyrosine kinase inhibitors (TKI), such as gefitinib and erlotinib, disrupt EGFR kinase activity by binding the adenosine triphosphate pocket within the catalytic region of the tyrosine kinase domain [9]. Currently, both gefitinib and erlotinib are used for treatment of patients with advanced NSCLC. TKI clinical trials have shown that these agents have dramatic effect on the subset of NSCLC patients with somatic mutations in the tyrosine kinase domain of the *EGFR *gene, whereas the presence of *KRAS *mutations seems to be correlated with primary resistance to these agents [10-15]. So it is necessary to identify the mutation status of *KRAS *and *EGFR *for selection of patients who are more likely to benefit from TKI. Although almost 70% of patients with NSCLC present with locally advanced or metastatic disease at the time of diagnosis [16,17], *KRAS *and *EGFR *mutation status is most commonly assessed only in the primary tumor tissue based on the assumption that primary and metastases are pathologically concordant. However, it has been known that lung cancers are often heterogeneous at the molecular level even within the same tumor and many key molecular alterations may occur during metastatic progression [18-20]. It is still unclear whether *KRAS *and *EGFR *mutation status in primary tumors is reflected in their corresponding metastases in Chinese patients with NSCLC, although several recent relevant studies in western countries have been performed and published [21-26].

In the present study, we investigate *KRAS *and *EGFR *mutation status using PCR-based sequencing analyses in 80 primary tumor samples and their corresponding local lymph node metastases from Chinese patients with NSCLC. The goal is to determine whether *KRAS *and *EGFR *mutation profile is stable during the metastatic progress and to investigate the clinical usefulness of mutational analyses in primary tumor versus in metastases for planning EGFR-targeted therapies for the treatment of patients with NSCLC.

## Materials and methods

### Patients and samples

Patients were selected from a pathological database of lung cancer cases undergoing curative resection for excision of primary tumor and the corresponding lymph nodes metastases at the Pathology Department of Tianjin Medical University Cancer Hospital from March 2009 to September 2009. Only patients with paraffin embedded tissues from surgically resected primary lung cancers and lung cancer-related local lymph node metastatic samples with histologically confirmed NSCLC were included. Patients who had been exposed to TKI before surgical treatment were excluded from this study. In each case, hematoxylin and eosin-stained sections of formalin-fixed paraffin-embedded tissue of primary tumor and corresponding synchronous lymph node metastases were reviewed by two pathologists to identify neoplastic areas and the amount of tumor cells in order to ensure that they contained more than 70% of tumor components for DNA extraction and mutation analysis. Tissue blocks were macro-dissected using a safety blade when samples were less than 70% of tumor cells. Primary tumor and lymph node specimens were obtained from all patients by surgical resection of primary tumors with lymph nodes dissection according to prevailing surgical standards. Consequently, 80 pairs of primary tumors and the corresponding lymph nodes metastases were analyzed. All samples were from patients of Chinese origin with NSCLC. The characteristics of the included patients were shown in Table 1.

**Table 1:** Patients' Characteristics (N = 80)

Characteristics	Patient Number (%)
Age, mean (range)	58 (32-77)
Gender	
Male	50 (62.5)
Female	30 (37.5)
Pathologic type	
Adenocarcinoma	39 (48.75)
Squamous cell carcinoma	31 (38.75)
Adenosquamous carcinoma	6 (7.5)
Large cell carcinoma	4 (5)
Smoking history	
Ever	49 (61.25)
Never	31 (38.75)

The inclusive criteria for selecting patients to receive gefitinib as neoadjunvant therapy were as follows: (1) NSCLC verified by cytology or histology; (2) age 18 to 70 years; (3) NSCLC with stage ⅢA or ⅢB and the tumors were confined in homolateral thoracic cavity; (4) patients without metastases in contralateral mediastinal lymph node; (5) patients who have never received treatment; (6) patients who could tolerate the surgery; (7) patients who were willing to receive preoperative target therapy. The exclusive criteria were: (1) without definite diagnosis; (2) age ≥ 70 years; (3) NSCLC with N3 or distant metastases; (4) small cell lung cancer; (5) patients who have been treated before; (6) patients who were unable to tolerate radical surgery. The local ethics committee granted approval, and written informed consent was obtained from each patient.

### DNA extraction

Thirty mg of frozen tissue was shredded by scissors. The E.Z.N.ATM Tissue DNA Kit (purchased by OMEGA) was used to extract genomic DNA. Quality and concentration of the DNA samples were examined by Nano Drop (Thermo™). Genomic DNA was then diluted to a working concentration of 5-10 ng/ul.

### PCR Amplification and sequencing

The two codons of *KRAS *(12 and 13) and two exons of *EGFR *(19 and 21) were amplified by PCR using the following forward and reverse primers: exon 1 of *KRAS*: 5'-AAAGGTACTGGTGGAGTATTTGATAGTG-3', 5 ' -TCATGAAAATGGTCAGAGAAACCT- 3 '; *EGFR *e x o n 1 9: 5 ' -AGCATGTGGCACCATCTCAC-3',5'-GCAGGGTCTAGAGCAGAGCAG-3'; *E G F R *e x o n 2 1: 5 ' - C T G A A T T C G G A T G C A G A G C T T - 3 ', 5 ' - C T A G T G G G A A G G C A G C C T G G T - 3. A total of 20 μl PCR reaction system included the following: 1x HotStarTaq buffer, 2.0 mM Mg2+, 0.2 mM dNTP, 0.2 μM of each primer, 1U HotStarTaq Polymerase (Qiagen), and 10ng DNA template. PCR reaction procedures were performed using 35 cycles of 15 sec at 94°C, 30 sec at 56°C, 1 min at 72°C and extension for 2 min at 72°C. Sequencing reactions were performed on an ABI3700 genetic analyzer after PCR products were purified. Sequence variations were determined using Seqscape software (Applied Biosystems) with the *KRAS *and *EGFR *reference sequence (NM_004985 and NM_005228.3, National Center for Biotechnology Information).

In order to avoid contamination during PCR steps, gloves and lab coats were worn at all times when PCR is performed. Pipette tips with aerosol filters were used to prevent microdroplets being injected into the PCR mixture. DNA sample preparation was done in a separate room from the area where PCR reaction mixes were prepared. Additionally negative control was also included during PCR procedure.

### Drug administration

Five patients received gefitinib as first-line treatment after being identified to harbor EGFR-TKI sensitive mutations in mediastinal lymph nodes metastases obtained by mediastinoscope. One tablet of gefitinib (250 mg) was taken once daily at about the same time. Patients continued the course uninterrupted until disease progression, intolerable toxicity or withdrawal of consent. All drugs were supplied by AstroZeneca.

### Assessment of response

Baseline evaluation included medical history and physical examination, electrocardiogram, chest radiography, thorax CT scan and ultrasonography of the upper abdomen. Laboratory investigations included complete blood counts, urinalysis, renal function and liver function tests. Performance status was evaluated according to the Eastern Cooperative Oncology Group (ECOG) criteria. Patients were re-evaluated, using the same method at the end of the first and third months of therapy, and then every 3 months. Objective tumor response and its duration were assessed according to the RECIST criteria [27], and all responses were confirmed >28 days after the initial assessment of response.

### Statistical analysis

McNemar's test was used to compare the *EGFR *and *KRAS *mutation status between primary tumors and corresponding local lymph node metastases. Two-sided *p *values <0.05 were considered significant. Data evaluation was carried out with SPSS_13.0 statistical software.

## Results

### *KRAS *gene mutations in NSCLC primary tumors and corresponding local lymph node metastases

*KRAS *mutations were detected in one primary tumor and seven lymph node metastases (Table 2). All of them were point mutations: five in codon 12 (G12A, G12V, G12S), two in codon 13 (G13D). Only one patient carried the same *KRAS *mutation in both primary tumor and metastatic tumor (Table 2, case 31). Six samples had mutations in lymph node metastases but not in their corresponding primary tumor tissues (Table 2, case7 to case12). Two of the *KRAS *mutation-positive samples (Table 2, case 7 and case 8) also carried the L858R *EGFR *mutation. NSCLC samples harboring both *KRAS *and *EGFR *mutations have rarely been reported previously. One sample had a *KRAS *mutation only in the metastases; the other one had *KRAS *mutations in both sites. The correlation between *KRAS *mutation and clinical parameters such as gender, smoke history and pathologic type was not statistically significant. Discordance in *KRAS *mutation status between primary tumors and lymph node metastases observed in six patients was found statistically significant (McNemar's test, *P *= 0.0412, Table 3). The majority (6/7) of all cases with *KRAS *mutations were squamous cell lung cancers. The other one was an adenocarcinoma.

**Table 2:** Comparison of *EGFR *and *KRAS *status between primary and metastatic tumors in NSCLC patients

Case No.	*EGFR *mutation status		*KRAS *mutation status	
	primary	metastasis	primary	metastasis
1	E746-A750	L747-T751	wt	wt
2	L747-P753insS	R748-P752	wt	wt
3	wt	L747-P753	wt	wt
4	wt	L858R	wt	wt
5	wt	L858R	wt	wt
6	wt	L858R	wt	wt
7	wt	L858R	wt	G12V
8	L858R	L858R	wt	G12A
9	wt	wt	wt	G12V
10	wt	wt	wt	G13D
11	wt	wt	wt	G12S
12	wt	wt	wt	G13D
13	E746-A750	E746-A750	wt	wt
14	E746-A750	E746-A750	wt	wt
15	E746-A750	E746-A750	wt	wt
16	E746-A750	E746-A750	wt	wt
17	E746-A750	E746-A750	wt	wt
18	E746-A750	E746-A750	wt	wt
19	E746-A750	E746-A750	wt	wt
20	L858R	L858R	wt	wt
21	L858R	L858R	wt	wt
22	L858R	L858R	wt	wt
23	L858R	L858R	wt	wt
24	L858R	L858R	wt	wt
25	L858R	L858R	wt	wt
26	L858R	L858R	wt	wt
27	L747-S752,P753E	L747-S752,P753E	wt	wt
28	E746-T751insV/A	E746-T751insV/A	wt	wt
29	E747-S752insV	E747-S752insV	wt	wt
30	I740-K745	I740-K745	wt	wt
31	wt	wt	G12A	G12A
32	wt	wt	wt	wt
.				
.				
.				
80	wt	wt	wt	wt

**Table 3:** Combined analysis of *EGFR *and *KRAS *status in NSCLC patients

Primary/Metastatic tumor					
	**WT/WT**	**WT/MUT**	**MUT/WT**	**MUT/MUT**	**Discordance**

*EGFR*	54	5	0	21*	7 case

*KRAS*	73	6	0	1	6 case

* E746-A750/L747-T751; L747-P753insS/R748-P752

Abbreviation: WT, wild type; MUT, mutational type

### *EGFR *gene mutations in NSCLC primary tumors and corresponding local lymph node metastases

*EGFR *mutations were detected in twenty-one primary tumors and twenty-six lymph node metastases. The types and locations of the mutations in paired tumors were shown in Table 2. Thirteen cases of the in-frame deletions in exon 19 and eight cases of point mutation in exon 21 were found in the primary tumors. Twenty-six cases with *EGFR *mutations in the lymph nodes included fourteen cases of the in-frame deletions in exon 19 and twelve cases of the point mutation in exon 21. All point mutations found in those samples were Leucin to Arginine at position 858 (L858R). The clinicopathologic characteristics that were significantly associated with EGFR mutations were gender, smoke history and pathologic type. Woman, non-smoker and adenocarcinoma showed a higher percentage of EGFR mutations (60%, 55% and 48%, respectively; *P *< 0.05). Discordant cases included five cases with no *EGFR *mutation in the primary tumors (Table 2, cases 3 to 7) and two cases with the metastases having a different *EGFR *mutation (Table 2, case 1 and case 2) (McNemar's test, *P *= 0.0736, Table 3).

### Response to gefitinib as neoadjuvant treatment

**Figure 1:**
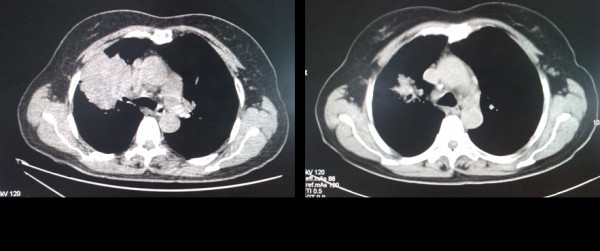
**Case 21 showed that the sizes of both the primary tumor and the mediastinal lymph nodes were found to shrink after gefitinib therapy when examined by thorax CT scan**.

**Figure 2:**
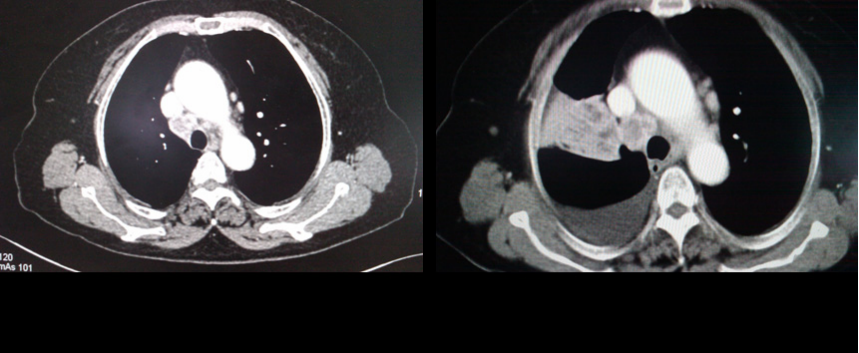
**Case 3 showed volume increase of primary tumor and obvious hydrothorax after gefitinib therapy, as determined by thorax CT scan**.

Five patients (Table 2, case 3 and cases 20 to 23) were given gefitinib as neoadjunvant treatment after the EGFR-TKI sensitive mutations were detected in their biopsies of mediastinal lymph nodes metastases by DNA direct sequencing. Of the five patients, three harbored delE746-A750 in exon 19 and the other two harbored L858R in exon 21. Four patients showed response to gefitinib and one experienced progressive disease. Among the four patients showing response to gefitinib, the size of both primary tumors and the mediastinal lymph nodes were found to shrink when examined by thorax CT scan (Figure 1). All four patients responded to gefitinib then received radical resection of the pulmonary carcinomas successfully after being evaluated to be suitable for surgery. Then their primary tumors harvested from surgery were examined for the *EGFR *mutations. We found that all four samples had the same mutations as those found in their mediastinal lymph nodes metastases. The patient who experienced progressive disease on gefitinib showed volume increase of the primary tumor and obvious hydrothorax, not a candidate for surgery according to NCCN Guidelines™ (Figure 2). With permission of this patient, we obtained his primary tumor tissue through ultrasound-guided aspiration in order to examine the gene mutation status. No mutations were detected in either the *EGFR *gene or the *KRAS *gene in the primary tumor from this patient.

## Discussion

NSCLC represents a major global health problem, but the introduction of a novel class of targeted anti-neoplastic agents, EGFR TKI, directed against EGFR has significantly changed the therapeutic options available for patients with NSCLC. Several studies have shown that activating *EGFR *mutations in exon 18, 19 and 21 are associated with a 75-95% objective response rate with EGFR TKI, whereas *KRAS *mutations are associated with a lack of sensitivity to these agents. However, of all patients with newly diagnosed NSCLC, 65-75% has advanced and unresectable disease. Up to half of patients with NSCLC develop metastases at the time of the initial diagnosis, and more patients eventually experience metastases in the course of their disease. *KRAS *and *EGFR *mutation status has been analyzed in primary tumors in the majority of the current studies, but it has been demonstrated that lung cancers are often heterogeneous at the molecular level, even within the same tumor. In addition, molecular characteristics may differ between primary tumor and metastases. The classical model for metastatic process suggests that most cells of a given primary tumor have low metastatic potential and only a few cells acquire enough somatic mutations to become metastatic [28]. Consequently, it is of primary importance to verify the degree of correlation between primary tumor and corresponding metastases with regard to *KRAS *and *EGFR *mutation status in order to select patients who will be most likely to benefit from the treatment with TKI.

In this study we assessed *KRAS *and *EGFR *mutation status in 80 pairs of NSCLC primary tumors and their corresponding local lymph node metastases to evaluate whether *KRAS *and *EGFR *mutation status changed during disease progression. We found that tumors metastasized to the lymph nodes did not always show the same gene status as their primary compartments. In our study, the discordance in *KRAS *and *EGFR *gene status was 7.5% (6/80) and 8.75% (7/80), respectively. To our knowledge, there have been several recent similar studies in western countries. For example, Kalikaki et al. reported that the discordance in *KRAS *and *EGFR *gene status between primary tumors and corresponding metastases was 24% and 28% in 25 patients with NSCLC, respectively [24]. Schmid et al. reported that the *KRAS *and *EGFR *gene status in primary tumors and lymph node metastases were discordant in 25 (26%) and 6 (6.25%) patients among 96 patients, respectively [26]. Monaco et al. compared 40 pairs of primary lung tumors with their metastases and found nine cases (22.5%) with a discordant *KRAS *status [21]. More recently, Cortot et al. performed mutant-enriched PCR (ME-PCR) to analyze *KRAS *gene status in primary tumors and their matched metastases. They found that the use of ME-PCR allowed a resolution of the discordance in 3 of the 6 cases by demonstrating the presence of low levels of mutant *KRAS *in lesions that were found negative by direct sequencing. Their data suggests that some gene discordance could be resolved by using techniques with increased sensitivity and that highly sensitive tools are required to identify biomarkers [29]. The difference between our findings with low discordant rate and those earlier studies might be due to different ethnic background of the patients studied. In western countries, *KRAS *mutation rate is high in NSCLC patients, especially in those with adenocarcinoma (30%-50%), but *EGFR *mutation rate is low (3%-8%). However, Asian patients with NSCLC harbor more *EGFR *mutations (30%-60%) and fewer *KRAS *mutation (4%-24%) than western patients [30-37]. Given that there are obvious genetic differences between somatic mutations in *KRAS *and *EGFR *genes in patients from Asia and western countries, it is very likely that changes of the mutation status during disease progression are different. Because relevant data about Chinese or Asian was not searched, further study should be performed to disclose the molecular mechanism.

Majority of the discordant cases in our study showed *KRAS *and *EGFR *mutations in the metastatic tumors rather than in their corresponding primary tumors (Table 2). This result suggests that the gene mutation status may change during metastases after diagnosis of the primary tumors. Although the molecular basis for this disparity is unclear, this information still has potential important clinical implications. This biological phenomenon of discordant gene mutations could partially account for the fact that some advanced NSCLC patients with apparent wild-type *EGFR *respond to EGFR TKI and other patients with well-known EGFR TKI-sensitive mutations in their primary tumors failed to respond to EGFR TKI. It is interesting that in our study we observed one case with delL747-P753 in mediastinal lymph nodes metastases showing progressive disease after gefitinib therapy. No *EGFR *mutation was found in its paired primary tumor. To our knowledge, this is the first study of the relationship between gene mutational status in both primary tumor and corresponding metastases and TKI responsiveness.

Moreover, several previous studies assessing the *KRAS *mutation status in primary tumors have suggested that *KRAS *mutation is uncommon in squamous cell carcinomas. Our data showed that the *KRAS *mutations were detected in the primary tumor of one adenocarcinoma and also in six metastatic tumors (five squamous cell carcinomas and one adenocacinoma), consistent with those previous reports. This result also suggests that the *KRAS *mutations might play an important role during metastases of NSCLC, especially squamous cell carcinomas.

Neoadjuvant or presurgical therapy is a novel therapeutic strategy that is now being investigated in the treatment of NSCLC. In part predicated on the success of this paradigm in other malignancies (such as colorectal, pancreatic, and urothelial cancers), presurgical therapy has the potential to provide real-time clinical feedback on the responsiveness of the patient's overall tumor burden to a given systemic therapy before committing the patient to what could be a highly morbid surgical procedure. Other potential benefits of this approach include local tumor down-staging, which may make subsequent surgical extirpation less morbid. In the case of locally advanced NSCLC, presurgical therapy may eliminate micrometastatic disease at its earliest stage, thus diminishing the risk of metastatic progression postoperatively. With the development and implementation of molecular targeted therapies that can meaningfully affect the biology of both primary tumors and metastases, the practice has largely been extended into the era of targeted therapy. In our study among five patients with EGFR TKI-sensitive mutations in mediastinal lymph node metastases, there were four patients who showed tumor regression in response to EGFR TKI and underwent surgery. These responses included dimension reductions in both primary tumors and mediastinal lymph nodes, suggesting tumor down-staging. Therefore, it is intriguing to consider the utilization of targeted therapies as an adjunct to make the "unresectable" become resectable. Neoadjuvant target therapy for NSCLC could potentially become a new treatment option for locally advanced and metastatic disease. On the other hand, we should not ignore the possibility that gene mutation status of primary tumors is different from that of their metastases when neoadjuvant target therapy is considered. If discordance between primary tumors and metastases is not evaluated before therapy, the patients may not benefit from the targeted therapies. Taken together, we propose that biopsies of both primary tumors and metastatic tumors of patients with advanced NSCLC, though difficult to obtain, should be pursued to ascertain the mutation status of key genes. This will allow clinicians to better understand gene mutation status and the biology of patient tumors, so that better treatment options can be selected based on tumor responsiveness to those available targeted therapies such as EGFR TKI.

## Conclusions

In summary, the substantial discordance of *KRAS *and *EGFR *mutation status between primary tumors and metastatic tumors may have therapeutic implications for EGFR-targeted therapy strategy. For NSCLC patients with metastases, determining the *KRAS *and *EGFR *mutation status in both primary and metastatic tumors may be critical for making meaningful decisions regarding the appropriate use of targeted therapies.

## Competing interests

The authors declare that they have no competing interests.

## Authors' contributions

ZZ, CW and BS designed the study; LS and QZ performed experiments; LS and HL analyzed data and prepared the Tables and Figures; LS and BS drafted the manuscript. All authors have read and approved the final manuscript.
